# Biomarkers and molecular endotypes of sarcoidosis: lessons from omics and non-omics studies

**DOI:** 10.3389/fimmu.2023.1342429

**Published:** 2024-01-04

**Authors:** Hong-Long Ji, Nan Mile S. Xi, Chandra Mohan, Xiting Yan, Krishan G. Jain, Qun Sophia Zang, Vivian Gahtan, Runzhen Zhao

**Affiliations:** ^1^ Burn and Shock Trauma Research Institute, Stritch School of Medicine, Loyola University Chicago Health Sciences Division, Maywood, IL, United States; ^2^ Department of Surgery, Stritch School of Medicine, Loyola University Chicago Health Sciences Division, Maywood, IL, United States; ^3^ Department of Mathematics and Statistics at Loyola University Chicago, Chicago, IL, United States; ^4^ Biomedical Engineering & Medicine, University of Houston, Houston, TX, United States; ^5^ Department of Medicine, Division of Pulmonary, Critical Care, and Sleep Medicine Yale New Haven Hospital and Yale School of Medicine, New Haven, CT, United States

**Keywords:** sarcoidosis, biomarker, endotype, omics, machine learning algorithms

## Abstract

Sarcoidosis is a chronic granulomatous disorder characterized by unknown etiology, undetermined mechanisms, and non-specific therapies except TNF blockade. To improve our understanding of the pathogenicity and to predict the outcomes of the disease, the identification of new biomarkers and molecular endotypes is sorely needed. In this study, we systematically evaluate the biomarkers identified through Omics and non-Omics approaches in sarcoidosis. Most of the currently documented biomarkers for sarcoidosis are mainly identified through conventional “one-for-all” non-Omics targeted studies. Although the application of machine learning algorithms to identify biomarkers and endotypes from unbiased comprehensive Omics studies is still in its infancy, a series of biomarkers, overwhelmingly for diagnosis to differentiate sarcoidosis from healthy controls have been reported. In view of the fact that current biomarker profiles in sarcoidosis are scarce, fragmented and mostly not validated, there is an urgent need to identify novel sarcoidosis biomarkers and molecular endotypes using more advanced Omics approaches to facilitate disease diagnosis and prognosis, resolve disease heterogeneity, and facilitate personalized medicine.

## 1 Background

Sarcoidosis is an inflammatory disorder characterized by granuloma formation in affected organs, most often in the lung (~90%) (1, 2). Scadding stage of sarcoidosis is therefore based on intrathoracic involvement. The most common organs involved are the lung, skin, eyes, liver, lymph nodes, salivary glands, bone/joints/muscle, spleen, nervous system, kidneys, sinuses, and heart. For example, lung sarcoidosis is featured by the presence of coalescing, tightly clustered, non-necrotizing granulomas, and is complicated by lung fibrosis in up to 20% progressive patients who account for 75% sarcoidosis-related deaths of respiratory causes (2–5). The etiology of sarcoidosis is still unknown since it was first described 1.5 centuries ago (1). The prevalence, presentation, prognosis, and triggering antigen are extremely variable (6). The outcomes of lung sarcoidosis are affected by age, gender, race, incomes, environment, lung morbidity, lung leucocyte infiltration, requirement of treatment, and genetic variants of associated genes (e.g., human leucocyte antigen class II (HLA class II), tumor necrosis factor α (TNF-α) and annexin XI (ANXA11), among others) (1, 2). However, there are no reliable biomarkers for predicting the propensity of sarcoidosis to progress to lung fibrosis (7).

The combination of advanced Omics techniques and machine learning algorithms holds the potential to facilitate mechanistic investigations and discovery of novel biomarkers for this intriguing illness. Molecular endotypes and biomarkers provide critical information for the development of new therapeutics. Several studies on the phenotypes of sarcoidosis have been published (8–10), but they are outside the scope of this review. In sharp contrast, only one study on the molecular endotypes of the disease has recently been reported to date (11). Biomarkers are widely applied to diagnosis, differential diagnosis, prognosis, treatment response, disease activity, severity assessment, chronicity evaluation, and the implementation of interventions. In this paper, we systematically review the identified biomarkers based on their clinical relevance. The clinical data were analyzed with differentiation analysis, correlation analysis, biomarker-specific tests, and machine learning algorithms. Recently, several publications have comprehensively reviewed sarcoidosis biomarkers identified using genomics (12–14). We will not reiterate this topic here. Instead, we will focus on the transcriptomics (and other Omics) studies published since 2018, as earlier studies have already been covered in a prior review (15).

## 2 Workflow of omics analysis for biomarkers and endotypes

**Figure 1:**
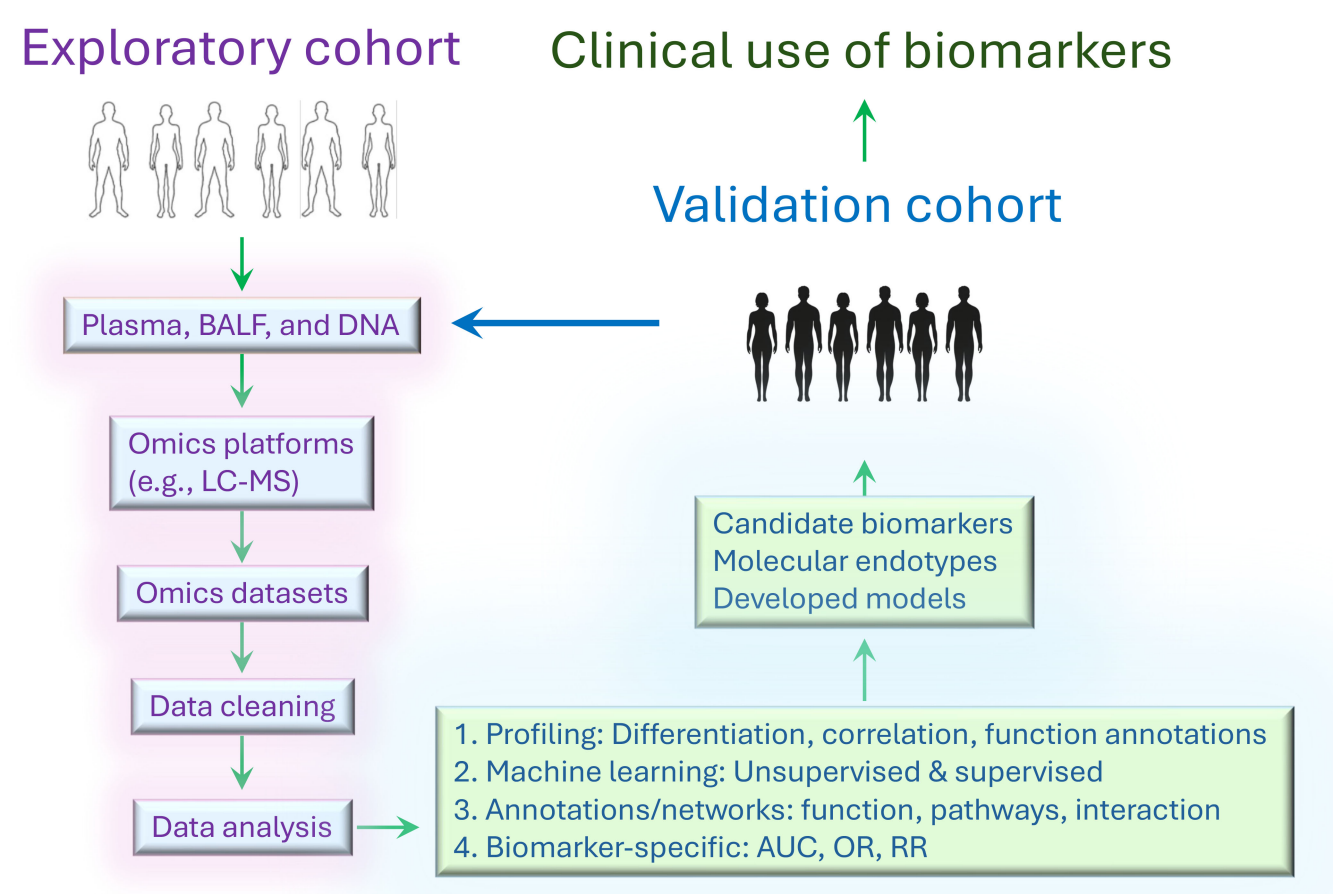
General workflow of Omics study to identify biomarkers and endotypes. First, liquid specimens and tissue samples collected from exploratory cohort are prepared ready for the analysis on the omics platforms. Second, omics datasets, including genomics, transcriptomics, proteomics, metabolomics, and microbiomics, are generated by experiments. Next, the datasets are pre-processed before statistical and machine learning analyses. Statistical analyses include differentiation expression tests, correlation analysis, logistic regression, Bayesian model, etc. Machine learning algorithms encompass supervised methods (e.g., gradient boosting, deep neural network, and laten classification and prediction) and unsupervised methods (e.g., PCA, k-means clustering, hierarchical clustering). Furthermore, network analysis can be performed with weighted gene co-expression network analysis (WGCNA), weighted protein correlation network analysis (WPCNA), genome-wide association study (GWAS) and meta-analysis. In general, function annotations are essential to rank differential gene-, transcript-, protein-, or metabolite-related signaling pathways, functions, and interactions. These analyses help identify candidate biomarkers, molecular endotypes, and unique phenotypes, along with developed predictive models. Specific metrics such as ROC AUC c statistic, odds ratio, risk ratio, and others are applied for biomarker comparisons. Finally, it is critical to validate the results in independent cohorts of multi-ethnic origins and under-represented populations, particularly since sarcoidosis is common in minorities.

The workflow of clinical omics studies is generally consistent, yet it adapts based on the specific objectives, materials, platforms, and data analysis strategies involved (**Figure 1**). Omics studies could be applied to the identification of candidate biomarkers, molecular endotypes, gene expression signatures, mechanisms, and druggable targets. The workflow is initiated by collecting relevant biologic specimens, including cells, tissues, and body fluid samples from sarcoidosis patients and healthy controls. The specimens are then pre-processed (e.g., obtaining single-cell suspension, extracellular vesicle isolation, or DNA/RNA/protein extraction) ready for the analysis by Omics platforms to generate Omics datasets. Once the datasets become available, data cleaning is required before downstream analysis, including normalization, missing data imputation, batch effect correction, and application of cut-off criteria. Next, the datasets will be profiled to identify differentially expressed genes/transcripts/proteins/metabolites. Various statistical and machine learning techniques are employed based on the specific tasks at hand. For instance, unsupervised clustering can be utilized to uncover novel molecular endotypes, while supervised logistic regression is appropriate for finding potential biomarkers. Omics studies can shed light on disease signatures, candidate biomarkers, molecular endotypes, and clinical disease-omics correlations. Finally, these observations will be cross-referenced to functional annotations, correlations with clinical variables, and literature reports. Validation of candidate biomarkers and molecular endotypes is also required in a separate independent cohort. An alternative strategy is to split one cohort into training and validation group. Besides, preclinical animal models of the disease and organ-on-chip could be used to validate the identified markers and mechanisms of action (16, 17).

## 3 Characteristics of clinical studies

### 3.1 Selection criteria of clinical studies to review

We searched the PubMed using sarcoidosis, biomarker, and endotype as input keywords. Only original clinical studies were included. The hits were subsequently categorized by the employed data analysis strategies, including classical statistics, AUC (area under curve), machine learning algorithms, and the utilization of omics platforms. We documented and compared the types of biomarkers, sources of specimens, sample sizes, statistical and machine learning methods used, performance metrics of identified biomarkers and endotypes, and validation procedures.

### 3.2 Types of biomarkers by purposes

Biomarker refers to a broad spectrum of biomedical features, including the specific analytes, anatomic features, pathophysiological characteristics, and pharmacologic responses to therapeutic interventions that can be measured accurately and reproducibly. The biomarkers of sarcoidosis can be categorized based on their clinical applications. “Diagnostic biomarkers” serve to differentiate sarcoidosis patients from healthy individuals. “Differential diagnosis biomarkers” aid in differentiating sarcoidosis from other similar conditions. Some biomarkers exhibit specificity for disease severity, inflammation, disease activity, and/or chronicity. “Prognostic biomarkers” are predictive for the outcomes of the disease, including mortality, lung fibrosis, and organ failure. Additionally, “predictive” biomarkers are indicative of treatment response and intervention efficacy (12–14, 18–25). As shown in **Tables 1**, **2**, the most common biomarkers reported for sarcoidosis are for diagnosis, followed by prognosis, organ involvement, and treatment response.

**Table 1:** Biomarkers associated or correlated with sarcoidosis, identified using non-Omics approaches.

Type of biomarkers	Tissue/assay/country	Cohort	Statistics	Biomarkers	Validation
Diagnostic (26)	BAL/ELISA/Greece	20 SA, 10 Ctl	t-test	IL12, IL18	No
Diagnostic (27)	Lung tissue, BAL/qPCR, gene chip/USA	6 SA, 6 Ctl	IPA, t-test	MMP-12 and ADAMDEC1	Yes. 11 SA, 11 Ctl
Diagnostic (28)	Serum/ELISA/Japan	81 SA, 33 Ctl	t-test	SPD for uveitis of SA	No
Diagnostic (29)	Blood/microarray/Germany	18 SA, 18 Ctl	R/Limma, Enrichment, Hierarchical average clustering, Spearman correlation, RF	Matrix metallopeptidase 14 for differentiating from TB	No
Diagnostic (30)	BAL, serum/ELISA/Slovenia	85 SA, 9 Ctl	t-test, Spearman correlation	Chitotriosidase	No*
Diagnostic (31)	BAL cells, blood T lymphocytes/qPCR, flow cytometry/Czech	50 PS, 23 Ctl	Mann-Whitney U test, Spearman correlation	AUF1, TIA and NCL mRNA in BAL cells, AUF1 and HuR in PBTLs	No
Diagnostic (22)	Blood & BAL Tfh cells/flow cytometry/Italy	13 SA, 12 Ctl	Kruskal-Wallis test, Mann-Whitney *U*‐test, χ^2^ test, Spearman correlation	CCR4-, CXCR3- and CXCR5-expressing Tfh subsets	No
Diagnostic (32)	Lymph node puncture fluid, BAL, blood/flow cytometry/China	31 SA: 17 active, 14 inactive	Student t-test, Mann-Whitney U test, Spearman correlation	LP, CD4/CD8 ratio in BAL	No*
Diagnostic (33)	EVs in BAL/flow cytometry/Italy	42 SA, 24 HP	Kruskal-Wallis test, χ^2^ test, Wilks’ lambda tests. agglomerative hierarchical clustering (AHC) analysis, PCA	CD11c, CD1c, CD209, CD4, CD40, CD44, and CD8 that shared with HP	Yes. 10 SA, 11 HP*
Diagnostic (34)	CSF/ELISA/Japan	20 NS, 14 Ctl	Welch’s t-test, Mann-Whitney U test, Spearman correlation, Wilcoxon signed-rank test	B-cell-activating factor (BAFF)	No
Diagnostic & response to therapy (35)	Serum/fluorimetry/Italy	694 SA, 101 Ctl	Clinical outcome scale, Pearson Mantel Cox test	Chitotriosidase for chronicity (sensitivity 57%, specificity 72%) and response to therapy (24.8%, 11%)	No*

The listed studies did not attempt to compute the biomarker potential to accurately distinguish sarcoidosis from controls. SA, sarcoidosis; Ctl, controls; CSF, Cerebrospinal fluid; NS, neurosarcoidosis; BAL, bronchioalveolar fluid; EVs, extracellular vesicles; PS, pulmonary sarcoidosis; HP, hypersensitivity pneumonitis; AHC, hierarchical clustering; PCA, principal component analysis; RF, random forest; IPA, ingenuity pathway analysis; BAFF, B-cell-activating factor. * reported by more than one study. Biomarkers from 4 of 10 studies have been validated.

**Table 2:** Sarcoidosis biomarkers identified in non-omics studies that have been assessed for their potential to accurately distinguish disease groups.

Type of biomarkers	Tissue/assay/Country	Cohort	Biomarker and statistics	Validation
Prognostic (36)	Serum/fluorometry/Spain	209 SA	RR 2.78 for absence of erythema nodosum 2.47 for hyperglobulinemia, 2.17 for chronicity	No
Diagnostic (37)	Serum/ELISA/Netherlands	144 SA: 73 untreated, 71 treated	sIL-2R (AUC=0.89, 82%, 94%) in untreated and (AUC=0.80, 64%, 88%) in total (both treated and untreated) patients to monitor lung severity	No*
Diagnostic & prognostic (38)	Blood/microarray/UK	21 SA, 20 Ctl	17-analyte biomarker model (AUC=0.96), 6-analyte panel (0.90), 14-biomarker panel (AUC=0.90) for SA	No
Diagnostic (39)	Plasma/ELISA/Japan	29 CS, 21 PH	NT-proBNP (AUC=0.91) for distinguishing CS from PH	No*
Diagnostic, predictive, & response (40)	Serum/ELISA/Italy	232 SA	Chitotriosidase (AUC=0.89, 89%, 93%) for diagnosis	No*
Prognostic (25)	BAL, blood/ELISA/Italy	30 SA	Panel of 6 markers for lung function (AUC=0.96, 100%, 96%)	Yes
Prognostic (41)	Urine/ELISA/Japan	31 SA: 17 active 14 inactive	U-8-OHdG (AUC=0.98, 88%, 93%) for predicting inflammation	No*
Prognostic (42)	Urine/ELISA/Japan	30 CS, 20 active 10 inactive	U-8-OHdG (AUC=0.87, OR=1.2) for predicting cardiovascular-related death	No*
Diagnostic (43)	Urine/ELISA/Japan	62 SA: 36 active, 26 inactive	U-8-OHdG (AUC=0.90, 89%, 83%. OR=1.7) for diagnosis of ventricular tachycardia	No*
Diagnostic (44)	Serum/ELISA/Japan	72 SA	sIL-2R (AUC=0.90, 100%, 78%) for 3+ involved organs, AUC=0.81 (80%, 62%) for BHL and/or lambda sign, AUC=0.83 (80%, 65%) for pulmonary involvement. ACE (AUC=0.83, 47%, 87%) and lysozyme (AUC=0.71, 53%, 83%) for pulmonary involvement	No*
Prognostic (45)	Plasma/ELISA/Japan	172 SA: 49 CS 123 non-CS	BNP (AUC=0.85, 85%, 68%) and PHA (AUC=7.8) for identifying cardiac sarcoidosis	No*
Diagnostic (46)	Blood/ELISA/Turkey	59 SA, 25 Ctl	sIL-2R (AUC=0.80, 75%, 74%) and OR=4.0, hs-CRP (AUC=0.95, 95%, 82%) and OR =47.2, ACE (AUC=0.87, 85%, 72%) and OR =4.4 for differentiating active from inactive SA	No*
Prognostic (18)	Serum/ELISA/Italy	74 SA	KL-6 (AUC=0.79, 78%, 85%) for fibrotic lungs	No*
Diagnostic (47)	CSF/microarray/Germany	23 NS, 115 Ctl	CSF sIL-2R (AUC=0.72 and 0.75) to differentiate neurosarcoidosis from MS and NINDs, respectively	No*
Diagnostic (48)	Serum/ELISA/Japan	37 SA	sIL-2R (AUC=0.76) predictor for EBUS-TBNA-based diagnosis	No*
Prognostic (49)	Serum/ELISA/Netherlands	121 SA, 70 Ctl	sIL-2R (OR=2.1) for predicting chronicity	No*
Diagnostic (50)	Serum, WBC/ELISA/Italy	24 SA, ILD 40, cHP 26	KL-6 (AUC=0.82), CRP (AUC=0.72), WBC (AUC=0.71) to discriminate from cHP and ILD.	No*
Diagnostic (51)	BAL cells/IHC/Japan	18 SA, 16 HP	Area, perimeter, radius ratio, and roundness of lymphocyte nuclei, AUC=0.69, 0.70, 0.61, and 0.62	No
Diagnostic (52)	Serum/microarray/USA	106 PS, 120 PS+, 101 Ctl	11 IgM autoantibody panel for SA (AUC=0.90), 8 IgM autoantibody panel to distinguish PS and PS+ (AUC=0.93)	3 cohorts*

SA, sarcoidosis; Ctl, controls; CS, cardiovascular sarcoidosis; PH, pulmonary hypertension; AUC, area under the curve; OR, odd ratio; RR, risk ratio; cHP, chronic hypersensitivity pneumonitis; ILD, Interstitial lung diseases; CS, cardiac sarcoidosis; PH, pulmonary hypertension; PHA, Cox proportional hazard analysis; sIL-2R, soluble IL-2 receptor; EBUS-TBNA, endobronchial ultrasound-guided transbronchial fine needle aspiration; KL-6, Krebs von den Lungen 6; hs-CRP high-sensitivity C-reactive protein; ACE, angiotensin-converting enzyme; BNP, B-type natriuretic peptide; U-8-OHdG, urinary 8-hydroxy-2-deoxyguanosine; BHL, bilateral hilar lymphadenopathy; NT-proBNP, N-terminal pro-brain natriuretic peptide; MS, multiple sclerosis. NINDs, noninflammatory neurologic disease. * reported by more than one study. 15 out of 19 studies have been validated.

Biomarker is generally identified combining biomedical tests and biostatistic/bioinformatic analyses. Biomedical measurements include ELISA, MRI, IHC, echocardiogram, flow cytometry, etc., as summarized in **Tables 1**–**4** for sarcoidosis. These data will then be analyzed using t-test, odd ratio, AUC, logistic regression, and machine learning algorithms based on the nature of data. Validation in a separate cohort is essential for any potential biomarker candidate. AUC is an acceptable approach to compute the sensitivity, specificity, and accuracy of individual biomarker or a biomarker panel. This is a common strategy to identify and evaluate the biomarkers of sarcoidosis in both Omics and non-Omics studies.

**Table 3:** Sarcoidosis Biomarkers identified in Omics studies that have been assessed for their potential to accurately distinguish disease groups.

Omics	Biosample	Cohort	Biomarker & Statistics	Validation
Transcriptomics (53)	Alveolar macrophages/microarray/Japan	107 SA, 89 Ctl	CTSS (AUC=0.80, sen 0.70, spe 0.78) for differentiating SA from other lung diseases	No
Transcriptomics (54)	Lung samples/RNAseq/ China	21 SA, 5 Ctl	PTGER4 (0.96), AKR1C1(0.91), PLA2G6(0.89), AKR1C3 (0.86), LTA4H (0.88), PLA2G7 (0.87), and combined 0.91 for diagnosis	No
Proteomics (55)	Serum/2-DE, MALDI-TOF-MS/Netherlands	35 SA, 35 Ctl	ACE 0.78 (sen 0.71, spe 0.71), sIL-2R 0.67 (sen 0.63, spe 0.57), α-2chain of haptoglobin (sen 0.74, spe 0.71)	No
Proteomics (56)	Serum & lung tissues/LC-MS, ELISA/China	64 SA, 99 Ctl	Amyloid A (AUC=0.76, sen 0.96, spe 0.53) for diagnosis	No
Proteomics (57)	BAL, sera/microarray/Sweden	40 SA, 49 Ctl	ZNF688 (AUC=0.79), ARFGAP1 (AUC=0.76) for diagnosis.	Yes
Proteomics (23)	Serum EVs/LC-MS/Japan	7 SA, 5 Ctl	CD14 (AUC=0.81), LBP (AUC=0.84), ACE (AUC=0.88), sIL-2R (AUC=0.88), ACE+CD14 (AUC=0.96), ACE+LBP (AUC=0.96), combined 4 markers (AUC=0.98) for diagnosis.	Yes. 46 SA, 10 Ctl
Metabolomic (58)	Saliva/NMR/France	24 SA, 45 Ctl	Six metabolites were altered in SA, including methanol/D, butyrate/D, lactate/U, acetate/U, and N-butyrate/U. Cross-validation AUC=0.87	Yes
Metabolomic (59)	Plasma/LC-MS/USA	67 SA: 31 LF	Discriminating metabolites involved collagen pathway metabolites, the arginine-proline pathway. p-coumaroylagmatine and palmitoylcarnitine are markers for LF. AUC=0.92 (73%, 93%) for separating lung fibrosis	No
Metabolomic (60)	Plasma/NMR/Canada	43 PS: 30 civilians, 13 veterans	Six metabolites and 33 elements differ between two groups. AUC=1.0 for both NMR and LC-MS (100%, 100%) and 0.97 (91%, 90%) for ICP-MS to differ two groups	No

SA, sarcoidosis; Ctl, controls; EVs, extracellular vesicles; LC-MS, Liquid chromatography–mass spectrometry; 2-DE, two-dimensional gel electrophoresis; MALDI-TOF-MS, Matrix-assisted laser desorption/ionization-time of flight mass spectrometry; AUC, area under the curve; CTSS, cathepsin-S.

**Table 4:** Other Omics-identified sarcoidosis biomarkers.

Omics	Tissue/assay/country	Cohort	ML or Software	Key results	Validation
Transcriptomics (61)	Tregs of PBMC and BAL/FACS, open array, PCR/Poland	45 PS	PCA, networks	SA-related miR-155, miR-223. PS have increased TLR/NOD signaling, intrinsic apoptosis, inflammation. Upregulated TLR2/MyD88 in different subpopulations of PBMCs	No
Transcriptomics (62)	BAL cells/Affymetrix and qPCR/Poland	12 SA	Hierarchical clustering, enrichment	BAL cells have increased mRNA for ribosome biogenesis and increased lymphocytes	No
Transcriptomics (63)	Cardiac tissue/snRNAseq/USA,	4 CS, 4 Ctl	Seurat, SCRNIC, GENIE3, AUCell, UMAP, RunPCA, RcisTarget, CLusterProfiler	GPNMB (transmembrane glycoprotein NMB) as a novel marker of multinucleated giant cells, additional macrophage populations. mTOR (mammalian target of rapamycin) pathway activation in HLA-DR^+^ and SYLT3^+^ macrophages is associated with proliferation	No
Proteomics (64)	BAL cells/2DE/Sweden	7 SA, 7 Ctl	PCA for clustering, OPLS	25 proteins for Fc-mediated phagocytosis and clathrin-mediated endocytosis pathways	No
Proteomics (65)	BAL macrophages/LC-MS/Sweden	8 SA, 6 Ctl	Multivariate models, OPLS-DA	15 protein panel for separating LS and non-LS. R^2 =^ 0.9, Q=0.85	No
Proteomics (66)	BAL, serum LS/LC-MS/Sweden	11 LS, 12 nonLR,12 Ctl	PCA, OPLS	Fcγ-regulation-associated factors for diagnosis, IgG related factors for phenotyping	Yes
Metabolomic (58)	Saliva/NMR/France	24 SA, 45 Ctl	OPLS	Six metabolites were altered in SA, including methanol/D, butyrate/D, lactate/U, acetate/U, and N-butyrate/U.	No
Metabolomic (59)	Plasma/LC-MS/USA	67 SA: 31 LF	Multivariate models, OPLS, PCA	Discriminating metabolites involved collagen pathway metabolites, the arginine-proline pathway. p-coumaroylagmatine and palmitoylcarnitine are markers for LF.	No
Metabolomic (60)	Plasma/NMR/Canada	43 PS: 30 civilians, 13 veterans	MetaboAnalyst, MetaBox, SIMCA-P, PCA, PLS-DA, OPLS-DA	Six metabolites and 33 elements differ between Vet and Civ.	No
Metabolomic (67)	Serum/NMR/Poland	40 SA	OPLS-DA	Exercise decreases fatty acids, triglycerides, and total cholesterol. Lipid profile as a prognosticator for lung function recovery	No.
Microbiome (68)	BAL/16S rRNAseq/India	8 SA	LEfSe for microbial markers, LDA=3	Corynebacteriales, Corynebacterium, and Neisseria.OTUID_476 for SA	No

SA, sarcoidosis; Ctl, controls; PS, pulmonary sarcoidosis; nonLR, non-lung fibrosis; PCA, principal component analysis; LDA, linear discriminant analysis; OPLS-DA, orthogonal partial least squares discriminant analysis; SCRNIC, single-cell regulatory network inference and clustering; LS, Löfgrens syndrome; Spe, specificity; sen, sensitivity. One of 11 studies was validated.

### 3.3 Sources of specimens for biomarker study

Albeit sarcoidosis being a systemic disorder, both local and circulating biomarkers have been explored. Common biopsy specimens include whole blood, plasma, serum, blood cells, tissues from different organs, and liquid biosamples (bronchioalveolar lavage fluid (BAL), joint fluid, spinal fluid, urine, and lymph node puncture fluid), along with their derivatives (cells, extracellular vesicles (EVs), DNA, microRNA (miRNA), etc.). Both Omics and non-Omics approaches have been reported. Targeted non-Omics methods include colorimetry, fluorimetry, enzyme linked immunosorbent assay (ELISA), Western blot (WB), qPCR (quantitative polymerase chain reaction), microarray, flow cytometry, and immunohistochemistry (IHC) analyses of targeted molecules. The non-targeted Omics studies, on the other hand, utilized gene chips, advanced arrays, RNA sequencing (RNA-seq), mass spectrometry, nuclear magnetic resonance (NMR), and 16S rRNA-seq (see Section 4), permitting a more comprehensive screen, in an unbiased manner. Omics approaches have the advantage of being comprehensive and unbiassed despite being more expensive and computation intensive.

## 4 Sarcoidosis biomarkers identified by targeted non-omics studies

Studies designed to identify diagnostic markers typically compare patients with healthy controls. For the identification of differential diagnosis biomarkers, other diseases often serve as control groups. In contrast, individuals with only sarcoidosis are required for the studies aimed to identify predictive biomarkers, biomarkers for the response to therapy, activity, chronicity, organ involvement, and severity of the disease, with longitudinal follow up in some cases. The average sample size across 30 non-omics studies is 88, ranging from 6 to 694. Only two studies included a separate validation cohort (27, 33).

We first review cross-sectional studies (**Tables 1**–**3**). Among the diagnostic biomarkers for distinguishing sarcoidosis from healthy control, the following have been independently validated: serum and CSF soluble interleukin-2 receptor (sIL-2R) (AUCs: 0.67 - 0.90) (23, 37, 44, 46–49, 55), urinary U-8-OHdG (AUCs: 0.87 - 0.98) (41–43), serum angiotensin converting enzyme (ACE) (AUC: 0.78-0.88) (23, 44, 46, 55), serum chitotriosidase (AUC: 0.89) (30, 35, 40), serum KL-6 (AUC: 0.79-0.83) (18, 50), serum CRP (AUC: 0.72-0.95) (46, 50), and serum BNP (0.85-0.91) (39, 45). In addition, several isolated reports of diagnostic biomarkers have also been reported, without independent validation: BAL IL12, IL18, MMP14, CTSS, serum amyloid A, ZNF688, ARFGAP1, CD14, LBP, a-2chain of haptoglobin, and PHA, with AUCs ranging from 0.76 to 0.84. The best performers among these include serum LBP (AUCs: 0.84) and CD14 (AUC: 0.81) (23).

Serum sIL-2R have been reported to be reflective of lung dysfunction, inflammation, multiple organ involvement including thoracic phenotype, and diagnosis of neurosarcoidosis based on cerebrospinal fluid (CSF) level (37, 44, 46–49). Urinary 8-hydroxy-2′-deoxyguanosine (U-8-OHdG) has been reported to predict inflammation in active patients, cardiac death, and ventricular tachycardia (41–43). Serum chitotriosidase has been validated for the diagnosis, chronicity, and response to therapy (30, 35, 40), Serum ACE has been proposed as a biomarker for identifying active parenchymal infiltration and pulmonary involvement (44, 46). Similarly, C-reactive protein (CRP) can serve as a biomarker to discriminate between inactive and active cases, and also distinguishing sarcoidosis from interstitial lung disease (ILD) and chronic hypersensitivity pneumonitis (46, 50). Krebs von den Lungen-6 (KL-6) is a biomarker of fibrotic lungs in sarcoidosis (18, 50), as reported by Bergantini and coworkers. B-type natriuretic peptide (BNP) is a diagnostic marker for cardiac sarcoidosis and a predictor for heart failure (39, 45). Besides protein biomarkers, CD4 and CD8 T-cells, and their respective ratios in BAL extracellular vesicles (BAL EVs) and lymph node puncture fluid can potentially function as diagnostic biomarkers (32, 33). The sensitivity and specificity of these biomarkers vary. Other biomarkers reported in one study but not yet cross-validated include neopterin, interleukin-12 (IL12), interleukin 18 (IL18), surfactant protein D (SPD), metallic metalloproteinase-14 (MMP-14), a disintegrin and metalloproteinase domain-like protein decysin-1 (ADAMDEC1), B-cell-activating factor (BAFF), C-C motif chemokine receptor 4 (CCR4), chemokine receptor 3 (CXCR3), chemokine receptor 5 (CXCR5), lymphocyte profile (LP), and those composite biomarker panels (22, 25–29, 31–34, 36, 38, 51, 52).

## 5 Omics-based identification of biomarkers for sarcoidosis

In recent years, Omics studies for the identification of biomarkers and molecular endotypes have been emerging, using a wide array of platforms. They apply both machine learning algorithms and traditional biostatistics for data analysis. The biomarkers identified by genome-wide association and transcriptomics studies have been comprehensively reviewed recently (15, 20, 69, 70). Thus, this review will specifically delve into transcriptomics studies since 2018. Finally, we review other Omics studies, including proteomics, metabolomics, and microbiomics.

### 5.1 Transcriptomics

RNA-seq has been employed to identify transcriptomic biomarkers in biopsied lung tissue (54). Notably, prostaglandin E receptor 4 (PTGER4), aldo-keto reductase family 1 member C1 (AKR1C1), phospholipase A2 group VI (PLA2G6), aldo-keto reductase family 1 member C3 (AKR1C3), leukotriene A4 hydrolase (LTA4H), and phospholipase A2 group VI (PLA2G7) mRNA levels exhibited potential significance for distinguishing sarcoidosis from healthy controls, each yielding an AUC value exceeding 0.85 (**Table 3**). A gene microarray analysis unveiled Cathepsin-S (with an AUC of 0.80) in alveolar macrophages as a potential differentiator between sarcoidosis and other lung diseases (53).

Other transcriptomic studies have attempted to differentiate sarcoidosis patients by clinical phenotypes. Unsupervised clustering analyses have been used to identify biomarkers associated with pulmonary sarcoidosis, cardiac sarcoidosis, and other phenotypes (**Table 4**). miR-155 and miR-223 were associated with pulmonary sarcoidosis. In addition, upregulated TLR/NOD (toll-like receptor/nucleotide-binding oligomerization domain) signaling, intrinsic apoptosis, and inflammatory pathways were uncovered in sarcoidosis patients, compared to healthy controls (61). A clustering of 12 patients suggested an increase in mRNA for ribosome biogenesis and lymphocytes in BAL cells in all patients (62). Moreover, GPNMB (transmembrane glycoprotein NMB) emerged as a potential biomarker for multinucleated giant cells associated with cardiac sarcoidosis (63). It is worth noting that these studies are limited by their small sample sizes, which pose challenges in identifying highly sensitive and specific biomarkers. Another concern is the lack of cross-validation. Consequently, further investigations using independent cohorts, along with the integration of machine learning algorithms and classical statistics (AUC), warrant consideration.

### 5.2 Proteomics

Proteins are the main vehicles of cellular function, and their abnormal alterations can result in organ disorders. A couple of proteomic studies of sarcoidosis have been reported. Serum zinc finger protein 687 (ZNF688), ADP-ribosylation factor GTPase-activating protein 1 (ARFGAP1), CD14, and LBP (lipopolysaccharide-binding protein) have been identified as validated biomarkers for differentiating sarcoidosis from healthy control (23, 57). By comparison, serum α-2 chain of haptoglobin and amyloid A present potential as diagnostic biomarkers but await validation. Clustering analyses, including principal component analysis (PCA) and oracle product lifecycle analytics (OPLA), have been applied to select proteomics-based serum biomarkers for identifying sarcoidosis (**Table 4**). Typically, these analyses cluster protein panels to differentiate controls and disease cases. For example, a 25-protein panel related to Fcγ-mediated phagocytosis and clathrin-mediated endocytosis exhibited diagnostic potential, as did panels involving regulation-associated factors (64). Furthermore, Fc-regulation-associated factors and IgG-related factors have been reported to be biomarkers for the presence of Lofgren’s syndrome, a distinct phenotype of sarcoidosis (65, 66). The sample sizes of the studies using LC-MS (liquid chromatography mass spectroscopy) were generally small, with fewer than 10 cases. The identified proteins and protein panels should be analyzed individually for the AUC value of each panel member. Moreover, targeted proteomics using antibodies or aptamers as ligands, which exhibit increased technical sensitivity, have barely been applied to the study of sarcoidosis. Given that global proteomics is time-consuming and resource-intensive, there is a long way to go before the identification of individual proteins for clinical use and the translation of proteomics biomarkers to the bedside.

### 5.3 Metabolomics

To date, four studies have analyzed blood and saliva metabolites in sarcoidosis (58–60, 67). Either LC-MS or NMR was used to detect metabolites. Notably, a saliva-based panel of six metabolites demonstrated the ability to differentiate sarcoidosis patients from healthy controls, yielding an AUC of 0.87 (58). Moreover, plasma p-coumaroylagmatine and palmitoylcarnitine were identified as differential diagnosis biomarkers for lung fibrosis, with their involvement extending to collagen and arginine-proline pathways (59). To compare veteran (military or other occupation) from civilian sarcoidosis patients, one study identified six differentially expressed metabolites and 33 trace elements (60). Metabolomics has also been applied to characterize lipid profile responses to exercise in sarcoidosis patients (67). Fatty acids, triglycerides, and total cholesterol were significantly reduced in patients on exercise regimen, suggesting the potential of using blood lipid profile as a prognosticator for recovery of lung function. Clearly, metabolomics is a powerful approach to identify potential biomarkers for sarcoidosis. More studies are warranted to validate the identified metabolic biomarkers and to identify additional biomarkers for diagnosis, differential diagnosis, activity, severity, chronicity of the disease, outcomes, and response to treatment.

### 5.4 Microbiome

Accumulating evidence suggests that the crosstalk between the gut microbiota and the lung, known as the gut-lung axis, is critical. The lung microbiota of sarcoidosis has been reviewed recently (71). The identification of microbial biomarkers for sarcoidosis is an emerging direction. To date, only one study has performed 16S rRNA-seq of BAL on 8 sarcoidosis cases aiming to identify microbial markers for diagnosis. This study identified three taxa as potential biomarkers in BAL specific to sarcoidosis: Corynebacteriales, Corynebacterium, and Neisseria.OTUID_476, each with a linear discriminant analysis (LDA) score greater than 3.0 (68). Nonetheless, these potential microbial biomarkers await confirmation and rigorous statistical analyses. In addition to lung microbiota, microbial markers should be identified from the gut or other involved organs in future studies.

## 6 Molecular endotypes

The identification of molecular endotypes for a given disease will shed light on disease pathogenicity, diagnosis, stratification, prognosis, and development of new personalized therapies. Currently, there is only one report on the “transcriptomic” endotypes of sarcoidosis (11). Four potential endotypes were identified by unsupervised analysis of RNA-seq data in BAL cells, including hilar lymphadenopathy with increased acute T-cell immune response, extraocular organ involvement with phosphatidylinositol-3-kinase (PI3K) activation pathways, chronic and multiorgan disease with increased immune response pathways, and multiorgan disease with increased IL-1 and IL-18 immune and inflammatory responses. These mRNA-based endotypes based on signatures from BAL cells await independent validation (72). In addition, a clinical trial has recently been registered to define the endotypes of CD4 T helper and T regulator cell in sarcoidosis (73). Clearly, molecular endotype studies of sarcoidosis is just in its infancy.

## 7 Conclusion

Despite decades of both basic and clinician research, our understanding of sarcoidosis remains limited. No specific interventions exist for systemic or single organ sarcoidosis due to the incomplete understanding of its pathogenicity. Consequently, there is an urgent need to find the molecular basis of various phenotypes and biomarkers. This pursuit is crucial for predicting long-term outcomes and responses to the therapy targeting the different manifestations of sarcoidosis. So far, a substantial portion (70%) of biomarkers identified through targeted non-Omics studies have been cross validated by different groups, various phenotypes, or distinct organ involvements (**Table 5**). These non-Omics-derived biomarkers may serve multiple purpose, including roles in diagnosis, differential diagnosis, prognosis determination, and assessment of disease activity, chronicity, and severity, and evaluation of therapeutic response. However, these focused studies need to be expanded to or fortified with larger, unbiased Omics based studies, in order to uncover improved biomarkers for this disease.

**Table 5:** Summary of identified biomarkers by non-Omics and Omics studies.

Study	Diagnostic biomarkers	Prognostic biomarkers	Drug Response biomarkers
Non-omics	*sIL-2R* (37, 44, 46, 47), biomarker panels (25, 38, 52), *NT-proBNP* (39), *KL-6 and CRP* (46, 50), *chitotriosidase* (40), *ACE and neopterin* (44, 46), *IL12 and IL18* (26), *MMP-12 and ADAMDEC1* (27), SPD for uveitis (28), MMP-14 (29), *chitotriosidase* (30), AUF1, TIA and NCL mRNA (31), *LP and CD4/CD8 ratio* (32), B-cell-activating factor (34, 45)	*U-8-OHdG* (41–43), *BNP* (45), *KL-6* (18), *chitotriosidase* (35), *sIL-2R* (48, 49)	*Chitotriosidase* (35)
Transcriptomics	Increased miR-155, miR-223, TLR/NOD signaling, intrinsic apoptosis, and inflammation (61), increased mRNA for ribosome biogenesis in BAL cells (62), GPNMB for CS (63), CTSS (53), transcript of PTGER4, AKR1C1, PLA2G6, AKR1C3, LTA4H, and PLA2G7 (54)	–	–
Proteomics	α-2 chain of haptoglobin (55), amyloid A (56), ZNF688 and ARFGAP1 (57), CD14 and LBP (23), protein panels (64–66)	–	–
Metabolomics	*Six metabolites* (58), p-coumaroylagmatine and palmitoylcarnitine (59)		Lipid profile (67)
Microbiomics	Corynebacteriales, Corynebacterium, and Neisseria.OTUID_476 (68)	–	–

All validated, high-quality biomarkers are highlighted in italic. sIL-2R, soluble interleukin 2 receptor; NT-proBNP, N-terminal pro–B-type natriuretic peptide; KL-6, Krebs von den Lungen-6; CRP, c-reactive protein; ACE, angiotensin converting enzyme; MMP, matrix metalloproteinases; SPD, surfactant protein D; TLR, toll-like receptor; NOD, nucleotide-binding oligomerization domain–containing protein; BAL, bronchioalveolar lavage; GPNMB, transmembrane glycoprotein NMB; CS, cardiac sarcoidosis; CTSS, cathepsin S; LBP, lipopolysaccharide-binding protein; U-8-OHdG, urinary 8-hydroxy-2-deoxyguanosine; BNP, B-type natriuretic peptide. All others are the gene names. Note: All independently validate biomarkers are in italics.

Very few individual biomarkers are identified by both Omics and non-Omics studies. It is most likely due to divergent approaches and tissues. Non-Omics studies measured one or few biomarkers at the protein level. In contrast, Omics studies profile the landscape of genes, transcripts, proteins or metabolites and identify a panel of top-ranked biomarkers. These Omics biomarkers shall be validated by other clinical studies independently. One challenge of implementing these biomarkers clinically is the difficulty of identifying these phenotypes at the bedside using the same approaches. It remains a question whether genomics, transcriptomics, and metabolomics biomarkers could be validated by proteomics. In addition, the organ specificity of identified biomarkers may lead to the inconsistency between identified Omics and non-Omics biomarkers. Of note, the organ specific biomarker could be applied to differentiate involved organs. All validated high-quality biomarkers are highlighted in italic in **Table 5**. These validated biomarkers are recommended for sarcoidosis. Without doubt, the combination of non-Omics and Omics assays will improve the identification of biomarkers in sarcoidosis.

One advantage of Omics studies is their high throughput capacity. This feature enables the identification of molecular endotypes in sarcoidosis and multiple biomarkers ranked by their importance. The development of new bioinformatics and machine learning algorithms holds significant potential for extracting more accurate or predictive information from Omics datasets, to prioritize critical biomarkers, meet clinical needs, and to identify molecular endotypes associated with different phenotypes. With respect to sarcoidosis, Omics studies are still in its infancy. These reported Omics studies allude to several potential biomarkers/panels, signaling pathways, and integrated networks, but the general paucity of sensitivity, specificity and accuracy comparisons, insufficient statistical power, and few cross-validations prevent rigorous conclusions from being drawn. Most of Omics-derived biomarker panels are not ready to be translated to bedside. More Omics studies and multi-Omic integrative investigations are needed to validate published biomarkers, and to identify more accurate biomarkers for sarcoidosis, using well annotated reference cohorts. Clinical trials are necessary to evaluate the clinical application of top-ranked biomarkers, each on an individual basis. To sum, there is an urgent need to identify novel sarcoidosis biomarkers and molecular endotypes to facilitate disease diagnosis and prognosis, resolve disease heterogeneity and facilitate personalized medicine.

## Author contributions

H-LJ: Conceptualization, Funding acquisition, Project administration, Resources, Supervision, Validation, Visualization, Writing – original draft, Writing – review & editing. NX: Writing – original draft, Writing – review & editing. CM: Writing – review & editing. XY: Writing – review & editing. KJ: Writing – review & editing. RZ: Writing – original draft, Writing – review & editing. QZ: Writing – review & editing. VG: Writing – review & editing.

## Glossary

**Table:**

ACE	Angiotensin converting enzyme
ADAMDEC1	A disintegrin and metalloproteinase domain-like protein decysin-1
AKR1C1	Aldo-keto reductase family 1 member C1
AKR1C3	Aldo-keto reductase family 1 member C3
ANXA11	Annexin XI
ARFGAP1	ADP-ribosylation factor GTPase-activating protein 1
AUC	area under curve
BAFF	B-cell-activating factor
BAL	Bronchioalveolar lavage
BNP	B-type natriuretic peptide
CCR4	C-C chemokine receptor type 4
CRP	C reactive protein
CSF	cerebrospinal fluid
CTSS	Cathepsin S
CXCR3	chemokine receptor type 3
CXCR5	chemokine receptor type 5
ELISA	Enzyme linked immunosorbent assay
EVs	Extracellular vesicles
GPNMB	Transmembrane glycoprotein NMB
GWAS	Genome-wide association study
HLA class II	Human leucocyte antigen class II
IHC	Immunohistochemistry
IL12	Interleukin -12
IL-18	interleukin 18
ILD	Interstitial Lung Disease
KL-6	Krebs von den Lungen-6
LBP	Lipopolysaccharide-binding protein
LC-MS	liquid chromatography mass spectroscopy
LDA	Linear discriminant analysis
LP	Lymphocyte Profile
LTA4H	Leukotriene A4 hydrolase
miR	microRNA
MMP-14	Metallic metalloproteinase -14
mRNA	messenger RNA
NMR	Nuclear magnetic resonance
NT-proBNP-	N-terminal pro-B-type natriuretic peptide
OPLA	Oracle Product Lifecycle Analytics
PCA	Principal Component Analysis
PI3K	Phosphatidylinositol-3-kinase
PLA2G6	Phospholipase A2 group VI
PLA2G7	Phospholipase A2 group VI
PTGER4	Prostaglandin E Receptor 4
qPCR	quantitative Polymerase chain reaction
RNA-seq	RNA sequencing
sIL-2R	Soluble interleukin 2 receptor
SPD	Surfactant protein D
TLR/NOD	Toll-like receptor/nucleotide-binding oligomerization domain
TNF-a	Tumor necrosis factor alpha
U-8-OHdG	urinary 8-hydroxy-2′-deoxyguanosine
WB	Western blot
WGCNA	Weighted gene co-expression network analysis
WPCNA	Weighted protein correlation network analysis
ZNF688	Zinc finger protein 687
